# BBOX1, LACC1, MMP7 and SSTR1 as common predictors in obesity and non-alcoholic fatty liver disease

**DOI:** 10.1016/j.gendis.2024.101310

**Published:** 2024-04-24

**Authors:** Chaoyuan Huang, Guoming Chen, Dongqiang Luo, Jiyuan Zheng, Nan Zhong, Danyun Li, Yang Luo, Peizhen Huang, Ning Wang, Yibin Feng, Yiyuan Zheng

**Affiliations:** a Department of Gastroenterology, The Second Affiliated Hospital of Guangzhou University of Chinese Medicine, Guangzhou, Guangdong 510120, China; b Department of Gastroenterology, Guangdong Provincial Hospital of Chinese Medicine, Guangzhou, Guangdong 510120, China; c School of Chinese Medicine, Li Ka Shing Faculty of Medicine, The University of Hong Kong, Hong Kong SAR 999077, China; d Clifford Hospital, Guangzhou University of Chinese Medicine, Guangzhou, Guangdong 511495, China; e The First School of Clinical Medicine, Guangzhou University of Chinese Medicine, Guangzhou, Guangdong 510405, China; f The University of Edinburgh, Edinburgh EH8 9LQ, United Kingdom; g Shanghai University of Traditional Chinese Medicine, Shanghai 201203, China; h Department of Gastroenterology, Shanghai Municipal Hospital of Traditional Chinese Medicine, Shanghai University of Traditional Chinese Medicine, Shanghai 201203, China

Obesity is one of the major risk factors of non-alcoholic fatty liver disease (NAFLD), however, the reliable diagnostic markers are not fully known. The purpose of this study is to clarify the shared pathophysiological diagnostic markers of obesity and NAFLD. We analyzed mRNA expression profiles of NAFLD and obesity from the GEO database using WGCNA to identify gene modules. Lasso regression and receiver operator characteristic curve helped construct predictive models. Key genes were examined for function via multiple analyses, including *in vivo* experiments and immune infiltration. *BBOX1*, *SSTR1*, *MMP7*, and *LACC1* emerged as common predictors for obesity and NAFLD through animal experimentation and predictive model analysis. Dendritic cells and macrophages were significantly altered in obesity and NAFLD (Fig. S1). Obesity and NAFLD share a molecular mechanism with *BBOX1*, *SSTR1*, *MMP7*, and *LACC1*, which play crucial roles in disease development and can serve as common predictors (Fig. S2, 3). Further research on drug–gene interplay may lead to new treatments.

Obesity is defined as abnormal or excessive body fat accumulation leading to impairment of health. Representing a worldwide major health challenge, obesity is associated with a decrease in life expectancy and is correlated with many metabolic diseases including liver disease and insulin resistance. The prevalence of NAFLD among adults worldwide is about one-fourth. With 20% of individuals developing cirrhosis, NAFLD has been linked to higher risks of both overall and liver-specific mortality.^1^ Obesity is one of the main risk factors for NAFLD, which is correlated with pro-inflammatory factors such as IL-1 and TNF. Over time, hepatic fat accumulation is promoted by a synergistic interaction between hepatic lipid dysregulation, oxidative stress, and pro-inflammatory cytokines, which results in the development of NAFLD.^2^ However, critical steps and valid diagnostic markers in the disease progress are incompletely understood. Therefore, our study constructed and evaluated the predictive models, consisting of four key genes for NAFLD and obesity that were elucidated from the GEO database, to further explore the mechanism between NAFLD and obesity as well as to offer new ideas for clinical treatment.

This study procured gene expression matrices of GSE162653 (obesity cohort) and GSE89632 (NAFLD cohort) from GEO (Gene Expression Omnibus, https://www.ncbinlm.nih.gov/geo/) and proceeded to perform unsupervised clustering analysis with the WGCNA package in R software. This resulted in the identification of 5 and 42 distinct gene modules, respectively. Notably, the turquoise and yellow modules of GSE162653 exhibited the strongest correlation with obesity (Fig. 1A), while the green and brown modules of GSE89653 were most highly correlated with NAFLD (Fig. 1B). These findings serve as the foundation for further analyses of the constituent genes of these modules. Moreover, genes exhibiting log_2_FC > 1 and *P* < 0.05 were identified as differentially expressed genes in patients with GSE162653 and GSE89632, respectively (Fig. 1C, D). The intersection of these genes with the selected module genes yielded the intersection genes (Fig. 1E).

**Figure 1:**
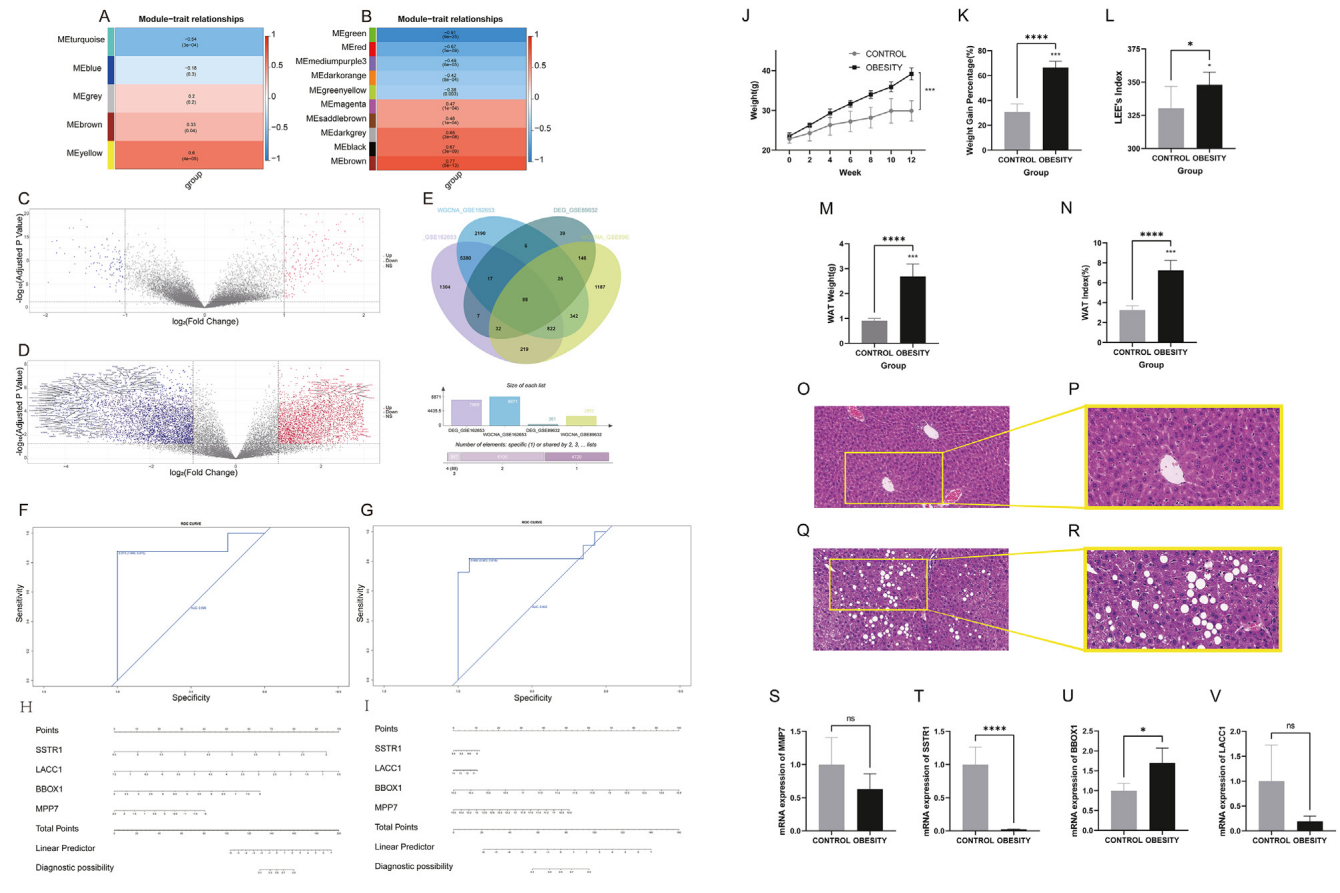
Screening of 4 key genes, model construction, and experimental validation. **(A, B)** Heat map of module–feature relationships for various soft threshold weights. **(C, D)** Volcano plot of the GSE89632 dataset and the GSE162653 dataset. The red dots represent up-regulated genes and the blue dots represent down-regulated genes. **(E)** Venn diagram. **(F)** Receiver operator characteristic (ROC) of obesity. **(G)** ROC of non-alcoholic fatty liver disease (NAFLD). **(H)** Nomogram of obesity. **(I)** Nomogram of NAFLD. **(J)** Weight tendency in 12 weeks. **(K)** Weight gain percentage. **(L)** Lee's index. **(M)** Weight of white adipose tissue (WAT). **(N)** WAT weight index. **(O)** Hematoxylin and eosin (H&E) staining (20 × ) for the control group. **(P)** H&E staining (40 × ) for the control group. **(Q)** H&E staining (20 × ) for the obesity group. **(R)** H&E staining (40 × ) for the obesity group. **(S)** Relative mRNA expression levels of *MMP7*. **(T)** Relative mRNA expression levels of *SSTR1*. **(U)** Relative mRNA expression levels of *BBOX1*. **(V)** Relative mRNA expression levels of *LACC1*.

Using Lasso regression analysis, we identified genes highly correlated with the diseases from the intersection genes. Specifically, 9 genes were found to be associated with obesity and 13 with NAFLD. Further intersection analysis revealed that *BBOX1*, *SSTR1*, *MMP7*, and *LACC1* were highly correlated with the onset of both diseases. We constructed prediction models for disease occurrence using these 4 genes in logistic regression analysis and performed receiver operator characteristic curve analysis, which revealed good predictive efficacy for both diseases (Fig. 1F–I).

To further investigate the roles of the 4 key genes in obesity and NAFLD, we conducted *in vivo* experiments in mice. We established a model for obesity and NAFLD in mice using a Western diet combined with high sugar solution, which showed significant differences in weight trend, weight gain percentage, Lee's index, WAT weight, and WAT index compared with the control group (Fig. 1J–N). Liver histology was performed to validate the diagnosis of NAFLD in mice. Hematoxylin and eosin staining revealed obvious hepatocyte steatosis in the obese mice model, indicating that obese mice co-existed with NAFLD (Fig. 1O–R). The PCR results revealed that the expression levels of *MMP7*, *SSTR1*, and *LACC1* were down-regulated, while the expression level of *BBOX1* was up-regulated in the experimental group (Fig. 1S–V).

Matrix metalloproteinase 7, also known as *MMP7*, plays a pivotal role in degrading various protein substrates in the extracellular matrix that regulates adipocyte production.^3^ Studies indicate that *MMP7* has the ability to attenuate its classical functions and antioxidant properties in lipid metabolism by cleaving apoA-IV, which may lead to obesity.^3^ Somatostatin receptor 1 (*SSTR1*) is mainly responsible for inhibiting the release of growth hormone.^4^ Interestingly, during the investigation of the SSTR1 inhibition of the growth hormone mechanism, it was observed that hyperinsulinemia was induced after knocking out the SSTR1 gene in mice, which is indeed one of the pathogenic mechanisms of NAFLD.^4^ Corresponding to our findings, research confirms that the mRNA expression of *BBOX1* is up-regulated in high-fat diet rats, and high-fat diets significantly affect the activity of *BBOX1* in rat liver.^5^ Currently, there are relatively few literature sources on *LACC1*. According to our research, *LACC1*-related genes are primarily enriched in metabolic pathways, such as the Hippo signaling pathway and PI3K-Akt signaling pathway, and *LACC1* expression is down-regulated in mice with obesity and NAFLD. These findings suggest that *LACC1* may promote the oxidative decomposition of fat and need further experimental validation.

In summary, *BBOX1*, *SSTR1*, *MMP7*, and *LACC1* are identified as diagnostic markers of obesity and NAFLD. Our clinical prediction model and *in vivo* experiments in mice confirm that these 4 genes are common predictors for obesity and NAFLD, and they likely play crucial roles in the development and onset of the diseases. Consequently, further research is necessary to explore the interplay between drug treatments and gene expression, potentially paving the way for new clinical treatment strategies.

## Ethics declaration

This study was approved by the First Affiliated Hospital of Guangzhou University of Chinese Medicine.

## Author contributions

YF and YZ contributed to the conception and design of the study. CH, GC, DqL, JZ, NZ, and DyL drafted the manuscript. YL and PH collected and analyzed the data. CH, NW, YZ, and YF revised the manuscript. All authors contributed to the manuscript revision and read and approved the submitted version.

## Data availability

The data of this study are available from the first authors upon reasonable request.

## Conflict of interests

The authors declared no conflict of interests.

## Funding

This study was funded by the 10.13039/501100001809National Natural Science Foundation of China (No. 82104549) and the 10.13039/501100002858China Postdoctoral Science Foundation (No. 2022M722158), and Shanghai Medical Innovation & Development Foundation (No. WL-YXBS-2022001K).

## Figures and Tables

**Fig. S1:**
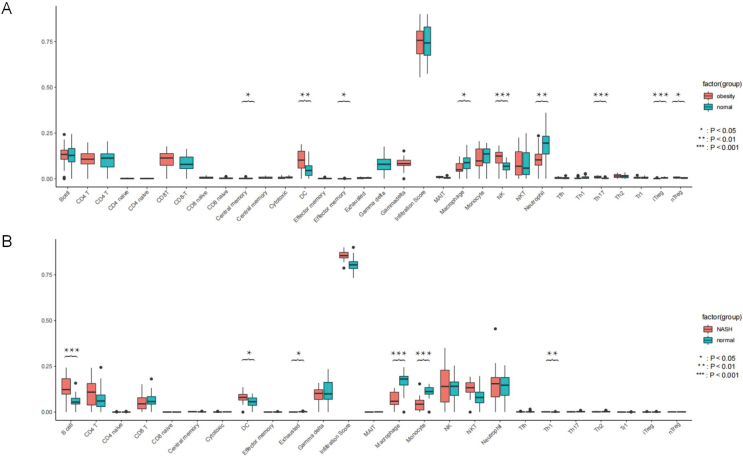


**Fig. S2:**
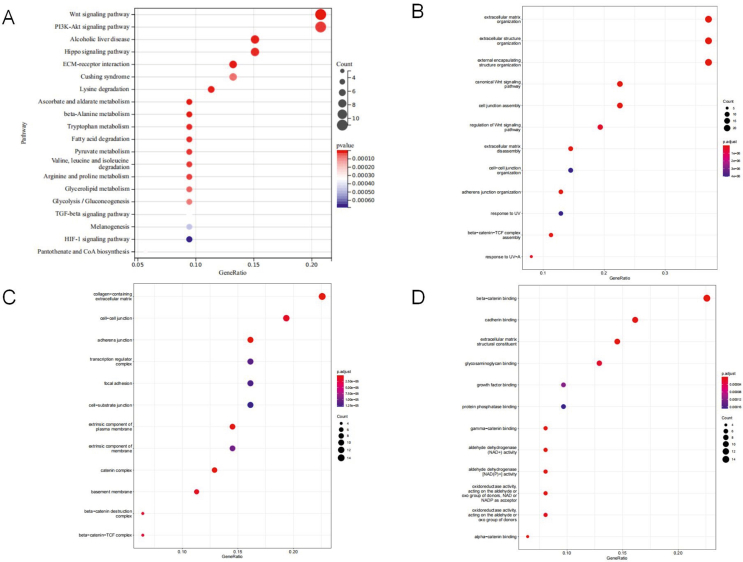


**Fig. S3:**
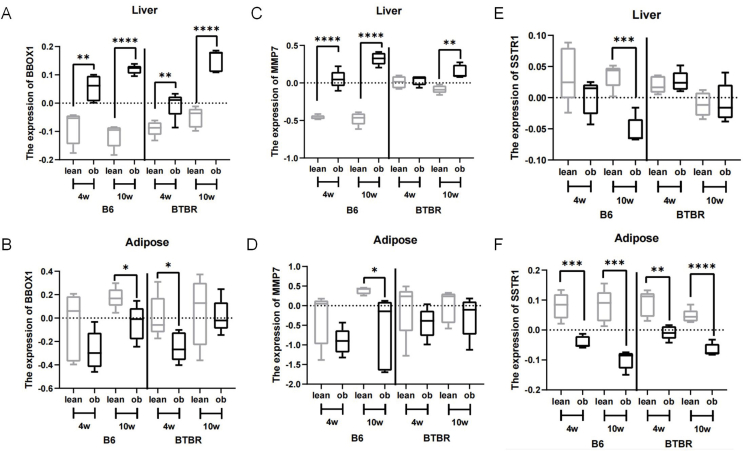


## References

[1] Sheka A.C., Adeyi O., Thompson J., Hameed B., Crawford P.A., Ikramuddin S. (2020). Nonalcoholic steatohepatitis: a review. JAMA.

[2] Dietrich P., Hellerbrand C. (2014). Non-alcoholic fatty liver disease, obesity and the metabolic syndrome. Best Pract Res Clin Gastroenterol.

[3] Grzechocińska B., Dąbrowski F.A., Sierdzinski J., Cyganek A., Wielgoś M. (2019). The association between serum metalloproteinase concentration, obesity, and hormone levels in reproductive-aged women. Endokrynol Pol.

[4] Kotronen A., Yki-Järvinen H. (2008). Fatty liver: a novel component of the metabolic syndrome. Arterioscler Thromb Vasc Biol.

[5] Rigault C., Le Borgne F., Tazir B., Benani A., Demarquoy J. (2013). A high-fat diet increases L-carnitine synthesis through a differential maturation of the Bbox1 mRNAs. Biochim Biophys Acta.

