# Supplementary material for: BBOX1, LACC1, MMP7 and SSTR1 as common predictors in obesity and non-alcoholic fatty liver disease
Source: Genes Dis. 2024 Apr 24;12(2):101310. doi: 10.1016/j.gendis.2024.101310 (PMC11605334; doi:10.1016/j.gendis.2024.101310)
Supplement: Multimedia component 1 [file mmc1.docx]

**Figure S1.** Differential Analysis of the Abundance of Immune Cell Infiltration. (A) Comparison of the abundance of immune cell infiltration between OB and normal samples. (B) Comparison of the abundance of immune cell infiltration between NASH and normal samples.

**Figure S2.** Biological Function Analysis of common predictors. (A) Kyoto Encyclopedia of Genes and Genomes enrichment bubble diagram. (B) Molecular Function enrichment bubble diagram. (C) Biological Process enrichment bubble diagram. (D) Cell Component enrichment bubble diagram.

**Figure S3.** Validation of the common predictors in database. These samples were obtained from B6 and BTBR mice. Both groups of mice were divided into ob mice and lean mice at 4 or 10 weeks of age. (A-B) Expression of BBOX1 in Liver and Adipose. (C-D) Expression of MMP7 in Liver and Adipose. (E-F) Expression of SSTR1 in Liver and Adipose.
