# Supplementary material for: BBOX1, LACC1, MMP7 and SSTR1 as common predictors in obesity and non-alcoholic fatty liver disease
Source: Genes Dis. 2024 Apr 24;12(2):101310. doi: 10.1016/j.gendis.2024.101310 (PMC11605334; doi:10.1016/j.gendis.2024.101310)
Supplement: Multimedia component 2 [file mmc2.docx]

**Table S1.** The top 20 diseases having comparable sets of interacting chemicals to NAFLD.

| Disease | Similarity Index | Common Interacting  Chemicals |
| --- | --- | --- |
| Dyslipidemias | 0.1779 | 37 |
| Hyperinsulinism | 0.1728 | 28 |
| Weight Gain | 0.1594 | 55 |
| Insulin Resistance | 0.1503 | 49 |
| Obesity | 0.1452 | 36 |
| Glucose Intolerance | 0.1397 | 32 |
| Liver Cirrhosis | 0.1331 | 39 |
| Fatty Liver | 0.1313 | 52 |
| Hyperlipidemias | 0.1239 | 28 |
| Hypercholesterolemia | 0.1228 | 28 |
| Diabetes Mellitus, Experimental | 0.1182 | 26 |
| Metabolic Syndrome | 0.1124 | 19 |
| Hyperglycemia | 0.1117 | 45 |
| Liver Cirrhosis, Experimental | 0.1111 | 27 |
| Fibrosis | 0.1056 | 34 |
| Diabetes Mellitus, Type 2 | 0.1053 | 24 |
| Hypertrophy | 0.1009 | 22 |
| Diabetes Mellitus | 0.0996 | 23 |
| Pancreatic Diseases | 0.0986 | 14 |
| Hypertriglyceridemia | 0.0985 | 20 |
