# Supplementary material for: BBOX1, LACC1, MMP7 and SSTR1 as common predictors in obesity and non-alcoholic fatty liver disease
Source: Genes Dis. 2024 Apr 24;12(2):101310. doi: 10.1016/j.gendis.2024.101310 (PMC11605334; doi:10.1016/j.gendis.2024.101310)
Supplement: Multimedia component 3 [file mmc3.docx]

**Table S2.** List of primer sequences used in this study.

| Primer Name | Sequence(5'to3') forward | Sequence(5'to3') reverse |
| --- | --- | --- |
| MUS-SSTR1 | CAGGGTAGCGCCATTCTCATC | AGCGTGGAAGTGACCAGAAAG |
| MUS-BBOX1 | ATGGGGCTCATTTGATGCAGA | GAAGTTTCCGAGCTTTTGCAG |
| MUS-MMP7 | GAGCAGTATGACACGGCAGAT | ACTCCGACAGGACCAACCA |
| MUS-LACC1 | CTGCCATGAGACCTTACTGGA | TTCCCGCGAGACGTGAAAAG |
| MUS-β-actin | GGGAAATCGTGCGTGAC | AGGCTGGAAAAGAGCCT |
